# Marine actinobacteria metabolites: unlocking new treatments for acne vulgaris

**DOI:** 10.3389/fmicb.2024.1501951

**Published:** 2025-01-06

**Authors:** María Clara De La Hoz-Romo, Luis Díaz, Javier Gómez-León, Marynes Quintero, Luisa Villamil

**Affiliations:** ^1^Doctoral Program of Biosciences, School of Engineering, Universidad de La Sabana, Chía, Cundinamarca, Colombia; ^2^Bioprospecting Research Group, School of Engineering, Universidad de La Sabana, Chía, Colombia; ^3^Marine Bioprospecting Line, Marine and Coastal Research Institute “José Benito Vives de Andréis” INVEMAR, Santa Marta, Colombia

**Keywords:** marine actinobacteria, acne vulgaris, antibacterial activity, secondary metabolites, *Cutibacterium acnes*

## Abstract

Marine-derived actinobacteria isolated from sponge *Cliona varians* and soft coral *Eunicea fusca* were screened for antibacterial activity against acne-related bacteria, specifically *Staphylococcus epidermidis* ATCC 14990, methicillin-resistant *Staphylococcus aureus* ATCC BAA44, and *Cutibacterium acnes* ATCC 6919. Cytotoxicity assays were performed on human dermal fibroblast (HDFa) and keratinocyte (HaCaT) cell lines to assess the safety profile of the extracts. Chemical characterization was conducted using high-performance liquid chromatography coupled with tandem mass spectrometry (HPLC–MS/MS). Among the extracts, six derived from *Kocuria* sp., *Rhodococcus* sp., *Nocardia* sp., *Micrococcus* sp., and *Streptomyces* sp. demonstrated significant antibacterial activity. Notably, extract Z9.216 from *Kocuria* sp. exhibited the highest efficacy, inhibiting *S. epidermidis* by 68%, *S. aureus* by 93%, and *C. acnes* by 98.7% at a concentration of 0.003 mg/mL, which was comparable to the standard antibiotics erythromycin and vancomycin, while maintaining over 90% cell viability in both HDFa and HaCaT cell lines. Untargeted metabolomic analysis suggested that antibacterial activity might be associated with compounds from the chemical families of alkaloids, terpenoids, and fatty acids, among others. These findings highlight the therapeutic potential of marine actinobacteria in underexplored environments as a promising strategy for treating acne vulgaris, a chronic inflammatory skin condition.

## 1 Introduction

Acne vulgaris is a multifactorial chronic inflammatory disease of the pilosebaceous follicle, which includes the hair shafts and sebaceous glands. It is the most common dermatological condition worldwide, with an estimated 650 million people (Moradi Tuchayi et al., 2015). Young people constitute the most compromised population (85%) (Melnik, 2018; Bernhardt and Myntti, 2016).

Disease severity is closely associated with the inflammatory response, mainly to *Cutibacterium acnes*, a prominent member of the skin microbiota. The skin microbiota is largely composed of Actinobacteria (Corynebacterineae and Propionibacterineae), Proteobacteria, Firmicutes (Staphylococcaceae), and Bacteroidetes (Mayslich et al., 2021). Certain phylotypes of *C. acnes* have been identified as opportunistic pathogens capable of causing invasive infections and forming biofilms (Mayslich et al., 2021; Achermann et al., 2014; Keshari et al., 2019). Additionally, bacterial interactions, such as those between *Staphylococcus epidermidis* and *C. acnes*, may be influenced by changes in host characteristics. These alterations can lead to the selection of pathogenic *C. acnes* strains that produce virulence factors, thereby increasing their inflammatory potential (Mayslich et al., 2021). Furthermore, shifts in the populations of *C. acnes* and *S. epidermidis* on the skin could promote colonization by *Staphylococcus aureus*, which is associated with acne vulgaris as well as other dermatological conditions, such as atopic dermatitis (Fournière et al., 2020).

Given the complexity of microbial interactions in acne, antibiotics are commonly used for its management. Nevertheless, they do not neutralize secretory toxins but instead exert selection pressure on non-target bacteria at a systematic level (Keshari et al., 2019). This has led to the emergence of resistance to erythromycin, clindamycin, and tetracycline in *C. acnes* and *S. aureus* strains, increasing the likelihood of treatment failure (Alkhawaja et al., 2020; Sermswan et al., 2023). This dynamic is particularly concerning in the context of bacterial resistance, as seen in *Staphylococcus aureus*. In 2019, methicillin-resistant *S. aureus* (MRSA) was responsible for an estimated 4.95 million deaths globally (Dadgostar, 2019). In addition, other treatment options for acne, such as isotretinoin, also produce side effects, including psychiatric events and inflammatory bowel disease (Costa et al., 2018).

Historically, natural products extracted from plants, animals, and microorganisms have been a prolific source of bioactive compounds. Nonetheless, these treatment options have been applied specifically to help stop the skin aging processes and pigmentation or improve the general appearance (Duarte et al., 2022; Bungau et al., 2023). Actinobacteria, in particular, are responsible for producing about 70% of currently used antibiotics (Dholakiya et al., 2017). Of the approximately 500.000 natural compounds are derived from biological sources, and 70.000 are of microbial origin, of which 29% are obtained from actinomycetes (Siro et al., 2022). Marine microbial communities have been reported as the most diverse source of biologically active compounds (Siddharth, 2019), and unique bioactive natural products have been reported from marine actinobacterial strains, such as marinopyrroles, heronapyrroles, ansalactam ammosamides, salinosporamide A6, and flavonoids, which were initially reported only in plants (Panche et al., 2016; Cheng et al., 2013). Furthermore, marine actinobacteria have been less explored than their terrestrial counterparts, providing an interesting field of study and a source of new bioactive compounds.

Three-quarters of all newly discovered bioactive microbial products in marine environments are produced by bacteria associated with marine invertebrates, and approximately 30% of these compounds originate from marine sponges (Rajasabapathy et al., 2020). Corals rank second after sponges in terms of productivity, with 5,800 compounds derived from corals accounting for nearly 20% of all natural marine products (Siro et al., 2022).

In this study, we explored extracts obtained from marine actinobacteria isolated from sponges and octocorals in the Colombian Caribbean. These extracts were obtained from 13 strains and were subjected to antibacterial analysis against *S. epidermidis*, *S. aureus,* and *C. acnes*. The promising extracts were further evaluated for cytotoxicity against human keratinocytes and fibroblast cell lines. Chemical analysis of the extracts was performed using a metabolomic approach. This analysis indicated that the compounds probably related to antibacterial activity belonged to the alkaloid, terpenoid, naphthalene, and stilbene families, among others, which have previously been reported to have significant antimicrobial potential. Furthermore, molecular identification of the strains producing these promising extracts revealed that most belong to a genera classified as rare actinobacteria, such as *Nocardia* sp., *Micrococcus* sp., *Rhodococcus* sp., and *Kocuria* sp.

These findings highlight the potential of marine actinobacteria as a promising source of bioactive compounds with significant antimicrobial properties, creating opportunities for the development of new therapeutic alternatives for treating acne vulgaris.

## 2 Materials and methods

### 2.1 Actinobacteria strains

Marine actinobacteria were obtained from the Microbial Collection of the Bioprospecting Research Group at Universidad de La Sabana, Colombia. These isolates were originally obtained from sponge and octocorals collected by scuba diving from the Colombian Caribbean, Bahía de Taganga, Punta Venado (11°16′23.9″ N, 74°12′24.9″ W), Bahía de Santa Marta, Punta Betín (11°15′02.1″ N, 74°13′16.0″ W), Magdalena, Colombia, at depths of 13 and 9 m, respectively (Sánchez-Suárez et al., 2021). The bacteria used in the present study were of Colombian origin and were obtained according to Amendment No. 5 to ARG Master Agreement No. 117 of May 26, 2015, granted by the Ministry of Environment and Sustainable Development, Colombia. Supplementary Table S1 describes the actinobacterial strains and their isolation sources.

*Staphylococcus epidermidis* (ATCC 14990), *C. acnes* (ATCC 6919), and methicillin-resistant *S. aureus* (ATCC BAA44) were acquired from the American Type Culture Collection (ATCC).

### 2.2 Actinobacteria culture and extracts obtention

Actinomycete isolates were inoculated in 100 mL of liquid Glucose, Yeast, and Malt extract broth (GYM) or Zobell Marine broth (Zobell), according to the original isolation medium (Sánchez-Suárez et al., 2021). Briefly, the culture started using a 55.81 mm^2^ plug from a 7-day-old lawn growth plate as a seed in 3 mL of broth. Each culture was incubated at 30°C with agitation at 200 rpm for 7 days. From this culture, 1 mL was used to inoculate 9 mL of GYM or Zobell broth, which was incubated for 7 days at 30°C with agitation at 200 rpm. Finally, the previous culture product (10 mL) was used to inoculate 90 mL of GYM or Zobell broth into 250 mL flasks on a rotary shaker (200 rpm) for 7 days. Then, 25 mL of each culture were placed in 50 mL plastic tubes, then freeze-dried (FreeZone Laboratory Freeze Dryer 2.5 liters, Labconco USA, Kansas), with a pressure of 0.22 millibar and a temperature of −55°C. The freeze-dried culture was transferred to a 50 mL Erlenmeyer flask, mixed with 10 mL of ethyl acetate (EtOAc), and agitated (150 rpm) for 24 h. The extraction was repeated three times using fresh EtOAc. The organic layers were evaporated under vacuum (Heidolph evaporator). Then, a stock concentration of 50 mg/mL was prepared for each actinobacteria extract, which was then used to perform antibacterial assays at the reported concentrations.

### 2.3 *Cutibacterium acnes* culture conditions

*Cutibacterium acnes* ATCC 6919 was activated according to the manufacturer instructions. Briefly, two media were used for its activation: ATTC Medium 2107: Modified Reinforced Clostridial (MRC) and ATCC Medium 260: trypticase soy agar/broth with defibrinated sheep blood under aseptic conditions and incubated in an anaerobic atmosphere at 37°C for 48–72 h. Anaerobic conditions were achieved using test tubes with a gas cannula system connected to anaerobic gas. Loose screw caps were placed in the test tubes in an activated anaerobic gas pack jar.

### 2.4 Antibacterial activity assays

The antibacterial activity of the extracts was estimated from the growth curve through the optical density using a Synergy H1 Multimode Reader (BioTek, Winooski, VT, USA). The antibacterial activity was determined using a microbroth susceptibility assay in 96-well plates, each containing 100 μL of extracts at different concentrations (initially 0.3, 0.03, and 0.003 mg/mL), following protocols standardized by the Bioprospecting Group of the Universidad de La Sabana, with slight modifications to the yield of the extracts (Sarmiento-Tovar et al., 2024). Extracts that did not show activity at the initial concentrations were further assessed at the highest concentration prepared (1.25 mg/mL), based on the yield of the extracts, with 100 μL of bacterial inoculum (*S. epidermidis*, MRSA, and *C. acnes*) at a cell density of $1.5\;x\;10^{8}$ UFC/mL. The absorbance was maintained within the range of 0.08–0.1 at 600 nm (Bauermeister et al., 2019). The optical density (OD) was measured at 600 nm at 30 min intervals for up to 18–20 h for *S. epidermidis* and MRSA. For *C. acnes*, the incubation time was 72–150 h the time at which the bacteria reached a stationary phase. The bacteria in Tryptic Soy Broth (TSB) and modified reinforced clostridial broth (MRC) were used as negative controls. The commercial antibiotics vancomycin and erythromycin, which were used at the same concentrations as the extracts, were used as the positive controls. Percentage inhibition was calculated using the following formula (Equation 1) (Rajivgandhi et al., 2016):


(1) $$\begin{array}{l} \%\;\mathrm{of}\;\mathrm{inhibition}:\\ \frac{\left(\mathrm{Untreated}\;\mathrm{bacteria}\;OD\;600\;\mathrm{nm}-\mathrm{Treated}\;\mathrm{bacteria}\;OD\;600\;\mathrm{nm}\right)}{\mathrm{Untreated}\;\mathrm{bacteria}\;OD\;600\;\mathrm{nm}}\times \;100 \end{array}$$

### 2.5 Determination of maximum specific growth rate and maximum OD

Specific growth rates were determined using ComBase^1^. This database contained statistical models that fit the nature of the data. The inhibition percentage was calculated using the following formula (Equation 2):


(2) $$\begin{array}{l} \%\;\mathrm{inhibition}=\\ \left(\frac{\mathrm{Untreated}\;\mathrm{bacteria}\;Gr^{1}-\mathrm{Treated}\;\mathrm{bacteria}\;Gr^{1}}{\mathrm{Untreated}\;\mathrm{bacteria}\;Gr^{1}}\right)\times \;100 \end{array}$$

^1^growth rate.

### 2.6 Minimal inhibitory concentration of bioactive extracts

The minimal inhibitory concentration (MIC) of extracts was determined using the broth microdilution method, according to the protocol described by Balagurunathan et al. (2020). Actinobacteria extracts and vancomycin and erythromycin (positive controls) were prepared by the serial 2-fold dilution method from the highest concentration observed in the antibacterial activity screening, 1.25 mg/mL (1.25–0.312 mg/mL) in TSB medium for *S. epidermidis* and *S. aureus*, and MRC broth for *C. acnes*. Freshly grown colonies of bacterial cultures were suspended in 10 mL fresh medium. The bacterial suspension was adjusted to a density of 5 × 10^5^ CFU/mL (Wang et al., 2017; Akhter et al., 2018) and added to 96-well plates. Each well contains 100 μL of the bacterial suspension and 100 μL of the extract. The negative controls included 100 μL of bacterial suspension and 100 μL of culture broth. As the extracts were dissolved in isopropyl alcohol (the maximum final concentration of isopropyl alcohol was 0.3%), a control with this solvent was also included. Each experiment was performed in duplicates. Plates were incubated for 20 h at 37°C for *S. aureus* and *S. epidermidis* and incubated for 150 h at 37°C under anaerobic conditions for *C. acnes* because of its naturally slower growth rate. MIC was determined by observing the lowest concentration of the extract that inhibited bacterial growth.

### 2.7 Cytotoxicity assay

Cell viability assays were performed to evaluate the cytotoxicity of the extracts against HDFa (Primary Dermal Fibroblast; Normal, Human, Adult ATCC® PCS-201-012™) and HACAT (spontaneously immortalized human keratinocyte cell line derived from a distant periphery of malignant melanoma) following the methodology described by van de Loosdrecht et al. (1991), with some modifications. Briefly, the cell lines were cultured in Dulbecco’s modified Eagle’s medium (DMEM) supplemented with 10% Fetal Bovine Serum (FBS) and 1% penicillin/streptomycin, using (3-(4,5-dimethylthiazol-2-yl)-2,5-diphenyltetrazolium bromide) (MTT) at 0.5 mg/mL an incubated 4 h. Cells (10^4^ cells/well) were seeded in triplicate in 96-well plates and cultured overnight at 37°C and 5% CO_2_. Bioactive extracts were added to 96-well plates at final concentrations of 0.3, 0.03, and 0.003 mg/mL and 1.25, 0.625, and 0.3125 mg/mL in DMEM supplemented with 10% fetal bovine serum. Dimethyl sulfoxide (DMSO, between 1 and 10% *v*/*v*) was used as the negative control. After 24 h of incubation, MTT was removed, and DMSO was added to each well to dissolve the formazan crystals. The amount of formazan was determined by measuring absorbance at 570 nm (Mosmann, 1983). Cell viability was calculated using the following formula (Equation 3) (Sánchez-Suárez et al., 2021):


(3) $$\%\;\mathrm{viability}=\left(\frac{OD_{\mathrm{Samples}}}{OD_{\mathrm{Control}\;\mathrm{group}}}\right)\times \;100$$

### 2.8 Sequencing of 16S rRNA gene and phylogenetic analysis

Genomic DNA was extracted using a Quick-DNA Fungal/Bacterial Microprep kit (Zymo Research Corporation, Irvine, CA, USA) according to the manufacturer’s instructions. The 16S rRNA gene was amplified using the universal primers 27F (forward primer: 5′-AGAGTTTGATCMTGGCTCAG-3′) and 1492R (reverse primer: 5′-TACGGYTACCTTGTTACGACTT-3′) under the following cycling conditions: denaturation at 94°C for 3 min, followed by 30 cycles of 94°C for 1 min, 50°C for 1 min, and 72°C for 2 min, with a final extension at 72°C for 7 min using Thermal Cycler (Bio-Rad). The amplified products were evaluated by electrophoresis of DNA samples on agarose gel. The agarose gel used in this study was 1% agarose gel in a buffer solution of Tris Acetate EDTA (TAE) (Anggelina et al., 2021).

The 16S rRNA sequences were run on the Basic Local Alignment Search Tool (BLAST) using the NCBI BLAST search tool, and the nearest neighbors were identified. MEGA version X (Molecular Evolutionary Genetics Analysis, version 10^2^), using the Tamura 3-parameter model, was used to construct a phylogenetic tree using the neighbor-joining method with a bootstrap test (1,000 replicates).

### 2.9 HPLC-QTOF-MS metabolomic analysis of extracts

Metabolomic analysis of the antibacterial active extracts and non-active extracts was performed using an Agilent Technologies 1,260 Liquid Chromatography system coupled with a Q-TOF 6545 time-of-flight quadrupole mass analyzer with electrospray ionization (ESI). The separation process was performed on a C18 column (InfinityLab Poroshell 120 EC-C18 100 × 3.0 mm, 2.7 μm). Two μL of the extracts were injected at 30°C and a gradient elution composed of: 0.1% (v/v) formic acid in Milli-Q water (Phase A) and 0.1% (v/v) formic acid in acetonitrile (Phase B) with a constant flow rate of 0.4 mL/min. The elution gradient program was: 0–15 min; 2–30% B, 15–17 min; 30–98% B, 17–21 min; 98% B and 21–26 min; 2%B.

Mass spectrometric detection was performed in the positive ESI mode in the full scan mode, and MS/MS was performed from 100 to 1.800 m/z. The following reference masses were used for mass correction during analysis: m/z = 121.0509 (C_5_H_4_N_4_) and m/z = 922.0098 (C_18_H_18_O_6_N_3_P_3_F_24_). The ESI source parameters were as follows: capillary voltage (3,000 V); drying gas (8 L/min); gas temperature (325°C); nebulizer pressure (50 psi); sheath gas temperature (350°C); and sheath gas glow (11 L/min). Q-ToF parameters included fragmentor voltage (175 V), skimmer voltage (65 V), and octapole radiofrequency peak-to-peak voltage (OCT RF Vpp) (750 V). Quality control (QC) samples were prepared according to the technique by pooling equal volumes of all groups of samples. QC pool samples were injected every 10 samples to evaluate stability and reproducibility throughout the analysis.

Data processing was performed using Agilent MassHunter Profinder software (version 10.0), which carried out deconvolution, alignment, and integration. These procedures were executed using the recursive feature extraction (RFE) algorithm. Datasets were filtered to remove features with a coefficient of variation (CV) > 20% in QC samples, retaining only those present in at least 80% of each sample group.

For metabolite identification, the CEU MASS MEDIATOR (accessible at https://ceumass.eps.uspceu.es/ as of August 15, 2023) (Gil-De-La-Fuente et al., 2019) was used to identify statistically significant m/z values. This tool integrates various platforms, including Metlin, Kegg, HDMB, and LipidMaps, with a tolerance of 10 ppm to annotate more abundant molecular features. Additionally, we utilized the StreptomeDB v3.0 (Moumbock et al., 2021) and the Natural Products Atlas v2.0 (Van Santen et al., 2022) databases.

To validate the identity of the metabolites, MS/MS analyses were conducted using MS-DIAL 4.8 (http://prime.psc.riken.jp/compms/msdial/main.html, accessed on August 22, 2023). These analyses involved *in silico* mass spectral fragmentation using CFM-ID 4.0 (available at https://cfmid.wishartlab.com/ as of August 28, 2023), and manual MS/MS spectral interpretation using the Agilent MassHunter Qualitative Analysis program (version 10.0, USA) (Camargo et al., 2023). The identified metabolites were reported according to the confidence level of compound annotation described by Blaženovi (2018).

### 2.10 Statistical data analysis

Data are presented as mean ± standard deviation (SD), and differences were examined using two-way analysis of variance (ANOVA) with a significance level of 5%. ANOVA assumptions of normality, homogeneity of variance, and independence of the data were checked. Dunnett’s test was performed to compare treatments at the 95% confidence level.

For metabolomic analysis, univariate statistical analysis (UVA) and multivariate statistical analysis (MVA) were performed to assess statistically significant differences among the metabolomic profiles of the groups using Metaboanalyst 5.0 and SIMCA 16.0 (Umetrics, Umea, Sweden), respectively. Initially, MVA based on principal component analysis (PCA) was applied to evaluate the quality of the acquired data. This ensured that the quality control (QC) samples were appropriately clustered in these models to guarantee the stability of the analytical system. Subsequently, orthogonal partial least squares discriminant analysis (OPLS-DA) models were constructed to maximize and examine the differences between the active and inactive extracts in the study groups and select the metabolites responsible for group separation.

Pareto scaling was applied for transformation before statistical analysis. In UVA, the *p-value* with a Benjamini-Hochberg false discovery rate correction (FDR) was determined (Quintero et al., 2022). Significant features were those with an adjusted *p-value* < 0.05, and variance important in projection (VIP) >1 with a jackknife confidence interval (JK). To determine the predictability and validity of the OPLS-DA model and to avoid overfitting and false positives, the models were subjected to cross-validation using K-fold and permutation tests (*n* = 100).

## 3 Results

### 3.1 Antibacterial activity of actinobacterial extracts

The antibacterial activity of ethyl acetate extracts from 13 actinomycete isolates was evaluated and established as a decrease in bacterial viability compared with that of untreated bacteria. Six extracts demonstrated activity against *Staphylococcus epidermidis*, MRSA, and *C. acnes*. The minimum inhibitory concentrations (MICs) were determined based on the concentrations tested. The Z9.216 extract exhibited MIC of 0.3 mg/mL against *S. epidermidis* and 0.003 mg/mL against both *S. aureus* and *C. acnes*. Extracts Z6.29 and Z9.11 had MIC values of 0.312 mg/mL for *S. epidermidis*, although MICs for *S. aureus* and *C. acnes* could not be determined within the tested concentration range due to the limited yield of the extracts. Table 1 shows the bacterial growth inhibition percentages of the extracts that were active against the three bacteria. These were selected for molecular identification by 16S rRNA gene sequencing. Biological profiling was carried out by metabolomic analyses of the end products using High-Performance Liquid Chromatography coupled with electrospray ionization quadrupole time-of-flight mass spectrometry (HPLC-QToF-MS) and mass spectrometry (MS/MS) analyses.

**Table 1:** Percentage of inhibition of bacterial growth by the most promising ethyl acetate extracts from marine actinobacteria against *Staphylococcus epidermidis* ATCC 14990, methicillin-resistant *Staphylococcus aureus* ATCC BA440, and *Cutibacterium acnes* ATCC 6919.

Extracts code	Extract concentration (mg/mL)	*S. epidermidis* ATCC 14990 (% of inhibition)	*S. aureus* ATCC BAA440 (% of inhibition)	*C. acnes* ATCC 6919 (% of inhibition)
Z9.216	0.003	68 ± 4.3^a^	93 ± 4.7^a^	98.7 ± 0.1^a^
G6.210	0.625	43 ± 0.0 ^a^	54 ± 2.0^a^	67 ± 6.1^a^
Z9.23	1.250	48 ± 5.6^a^	50 ± 3.4^a^	68.2 ± 7.5^a^
Z6.29	0.312	98 ± 3.5^a^	42 ± 5.6^a^	76.7 ± 4.0^a^
Z9.21	0.625	20 ± 2.0^a^	19 ± 3.8^a^	71 ± 8.0^a^
Z9.11	0.312	100 ± 0.7^a^	23 ± 0.2^a^	80 ± 4.0^a^

Values are expressed as mean ± standard deviation (*n* = 2). The minimum concentration at which each extract showed activity is reported. ^a^ Standard deviation (±SD) of two experiments.

**Figure 1:**
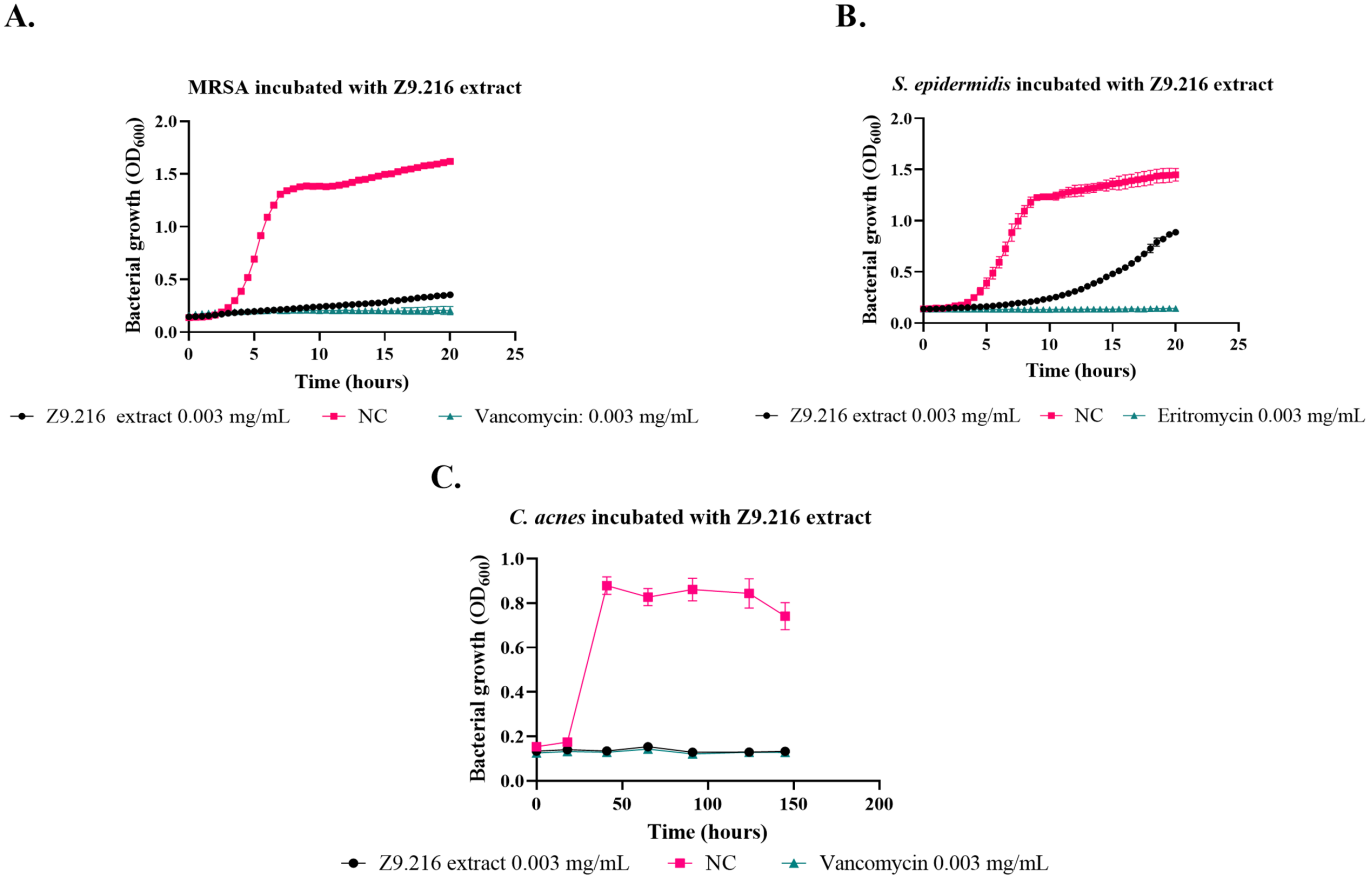
Effect of Z9.216 extract (0.003 mg/mL) on the growth of *S. epidermidis* ATCC 14990, methicillin-resistant *S. aureus* (MRSA) ATCC BA440, and *C. acnes* ATCC 6919. **(A)** Growth curve of *S. aureus* ATCC BA440; **(B)** Growth curve of *S. epidermidis* ATCC 14990; **(C)** Growth curve of *C. acnes* ATCC 6919. NC: Negative control, untreated bacteria.

The most promising isolates were selected because they exhibited more than 50% growth inhibition against some of the bacteria evaluated, namely Z9.216, G6.210, Z9.23, Z6.29, Z9.21, and Z9.11. The extract with the highest percentage of growth inhibition, Z9.216, was selected for further studies. Figure 1 presents the bacterial growth curves of MRSA, *S. epidermidis*, and *C. acnes* in the presence of Z9.216 extract.

### 3.2 Determination of maximum specific growth rate

**Figure 2:**
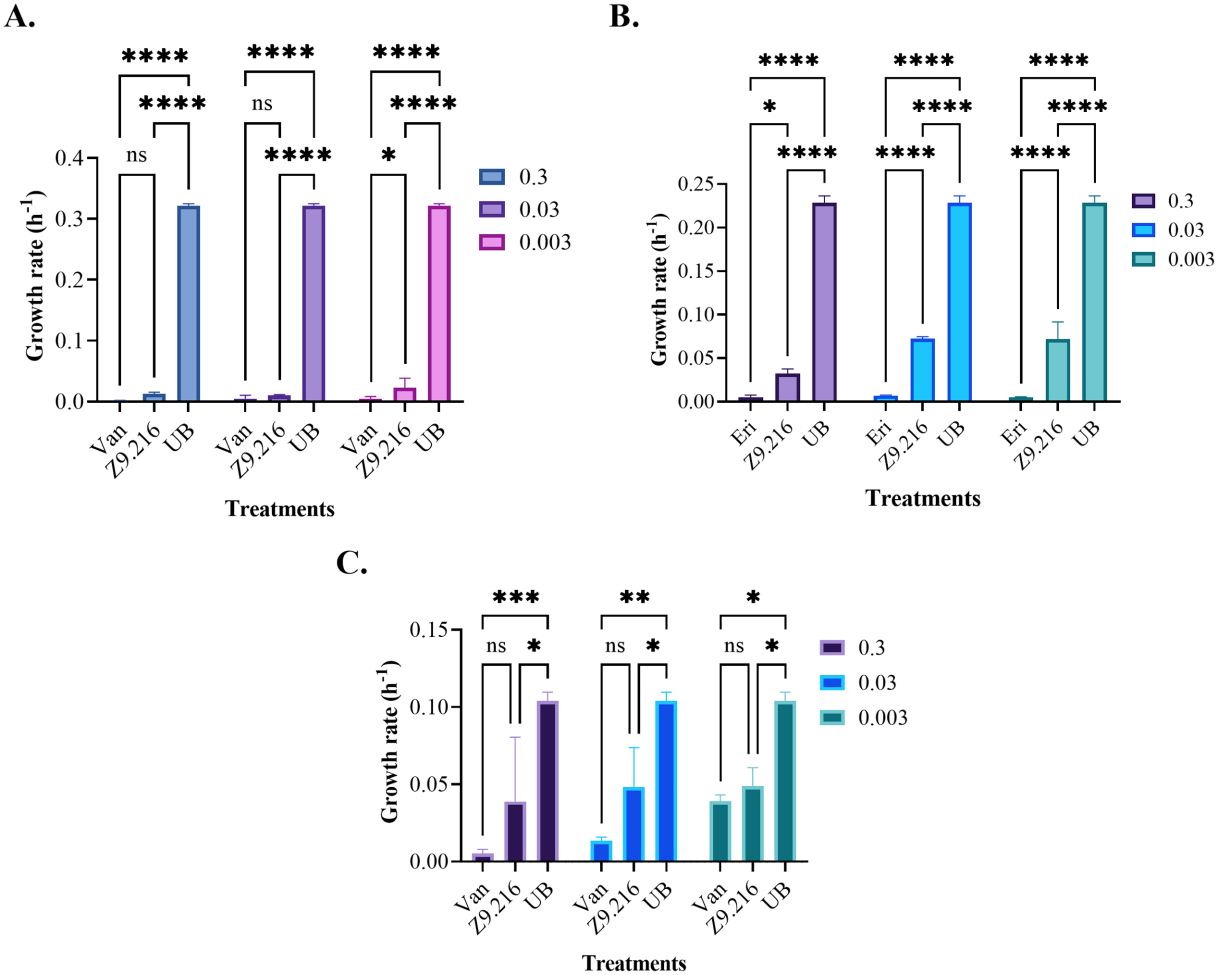
Growth rates of the bacteria studied in the presence of the Z9.216 extract (0.3–0.003 mg/mL). **(A)** Growth rate of methicillin-resistant *S. aureus* (MRSA) ATCC BA440. **(B)** Growth rate of *S. epidermidis* ATCC 14990. **(C)** Growth rate of *C. acnes* ATCC 6919. Values are expressed as mean ± standard error of the mean (SEM) and are compared with the positive control group. Significance levels are indicated as follows. ****p-value < 0.0001; ***p-value < 0.0007; **p-value < 0.0015; *p-value < 0.05.

The growth rate of bacteria treated with various extracts was assessed using the Baranyi and Roberts predictive primary model, because it had the best *R*^2^ parameters (>97%) and the lowest squared error (<0.2). The maximum growth rate (measured in units of hours (h^−1^)), represented by the *μ* parameter, indicating the exponential growth phase of the bacteria (Dalgaard et al., 1994; Lindqvist and Barmark, 2014), was determined based on this model. Analysis of the data revealed that the bacterial growth rate in the presence of the most promising extract, Z9.216, at concentrations of 0.3, 0.03, and 0.003 mg/mL closely resembled that of the antibiotic vancomycin against MRSA and erythromycin against *S. epidermidis* and *C. acnes*. Interestingly, bacteria tested in the presence of extracts G6.210, Z9.23, and Z6.29 at 1.25 mg/mL exhibited growth rates surpassing those of the antibiotics but falling short of the growth rate observed in untreated bacteria. In comparison, the performances of extracts Z9.21 and Z9.11 were found to be similar. Figure 2 illustrates the growth rates of the studied bacteria in the presence of the most promising extract, Z9.216.

### 3.3 *In vitro* safety evaluation of the actinobacterial extracts

**Figure 3:**
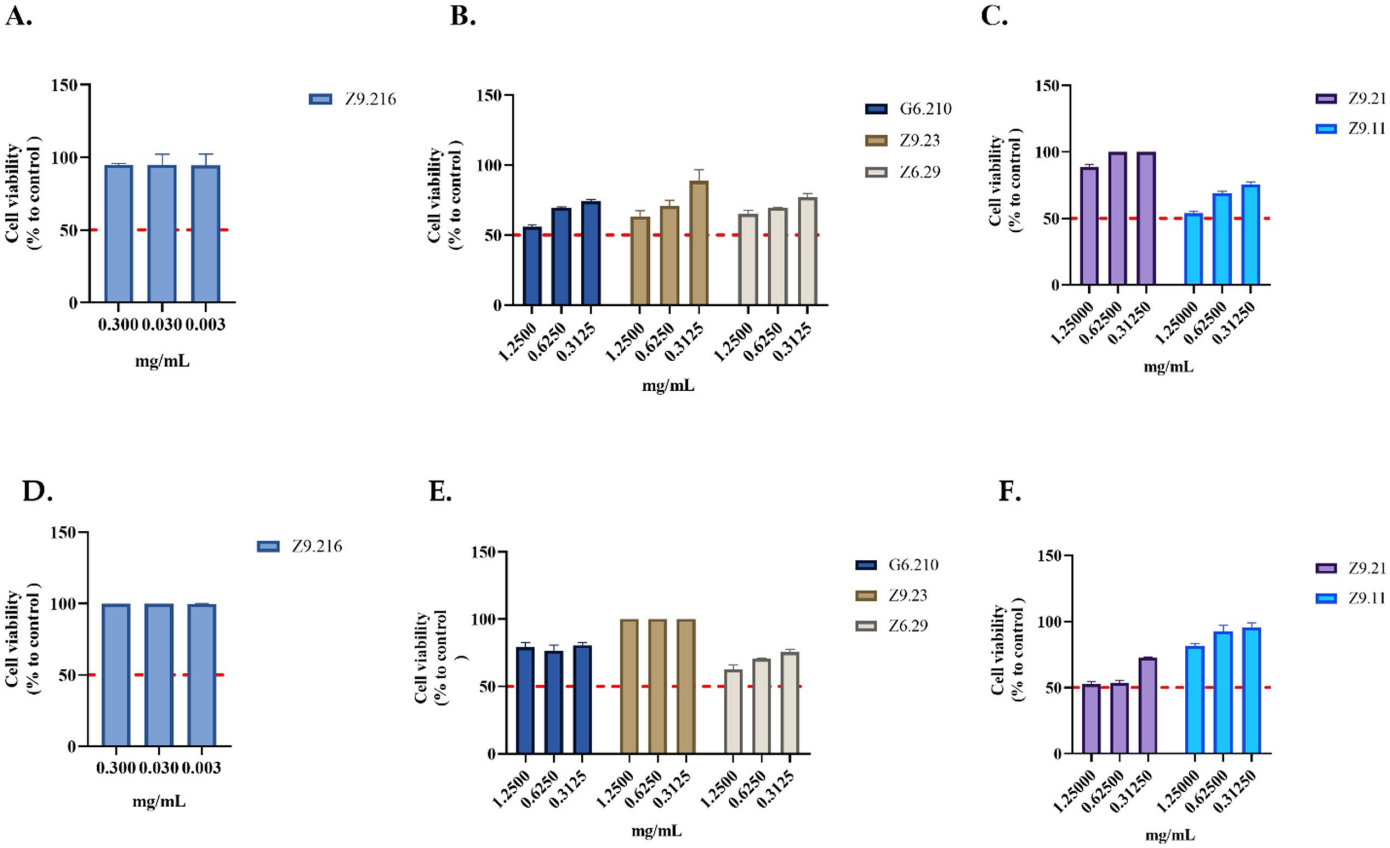
Evaluation of the viability of actinobacterial extracts. **(A–C)** Extracts evaluated in the HACAT cell line. **(D–F)** Extracts evaluated in HDFa cells. The crude extracts were evaluated at concentrations that showed activity. Each value represents the mean (*n* = 2) and error bars represent the standard deviation (SD). Cell viability was calculated by comparing the viability of untreated cells (control).

The most promising extract, Z9.216, maintained cell viability above 90% in both the HDFa and HACAT cell lines. In contrast, the bioactive extracts G6.210, Z9.23, and Z9.11, exhibited the highest cytotoxic activity against the HACAT cell line, reducing viability by 44, 36, and 46%, respectively, at the highest concentrations evaluated. In the HDFa cell line, the extracts Z6.29 and Z9.21 demonstrated the highest cytotoxicity, decreasing cell viability by 37 and 47%, respectively, at the highest concentrations tested. For the remaining actinobacterial extracts, cell viability remained above 70% (Figure 3).

### 3.4 Identification of isolates with antibacterial activity by 16S rRNA gene sequencing

**Figure 4:**
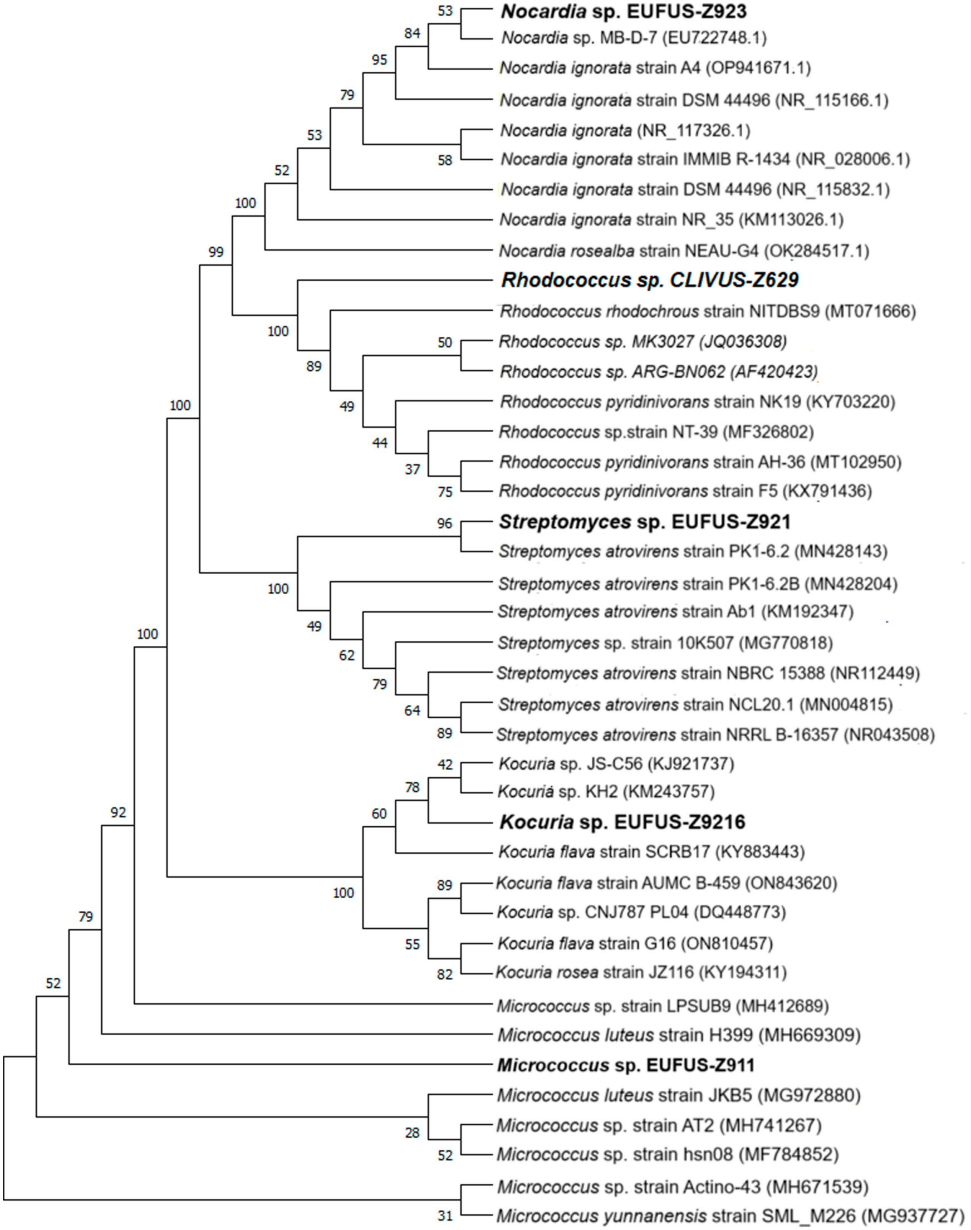
Phylogenetic tree of the bioactive isolates: cladogram for strains of genera *Kocuria* sp., *Nocardia* sp., *Rhodoccoccus* sp., *Micrococcus* sp., and *Streptomyces* sp., using 16S rDNA sequences. An optimal tree is shown. The accession codes for the blasted strains are shown in parentheses. The percentage of replicate trees with associated taxa clustered together in the bootstrap test (1,000 replicates) is displayed next to the branches. All ambiguous positions were removed from each pair of sequences (i.e., pairwise deletions).

The cladogram led to the classification of bioactive isolates among actinobacterial genera, and Z9.216 was identified as a *Kocuria*, Z9.23 as *Nocardia*, Z6.29 as *Rhodococcus*, Z9.11 as *Micrococcus*, and Z9.21 as *Streptomyces* (Figure 4). The G6.210 isolate belonged to the *Streptomyces* genus, as previously reported (Sánchez-Suárez et al., 2021). Consensus sequences of the remaining extracts were deposited in GenBank under accession numbers PP389604, PP741801, PP741804, PP741802, and PP741803. The coding of the end-products and the names of the identified strains are presented in Supplementary Table S1.

### 3.5 Untargeted metabolomics by HPLC-QTOF-MS of promising microbial extracts

A total of 468 features were obtained from the analysis, and the comparison between the antibacterial active extracts and non-active extracts yielded 45 statistically significant features. Among these, only 16 were annotated based on the criteria described by Blaženovi (2018). Furthermore, the retention time was considered to match the nature of the compounds for all the features. To assess the quality of the analytical platform, PCA was performed for each analysis.

**Figure 5:**
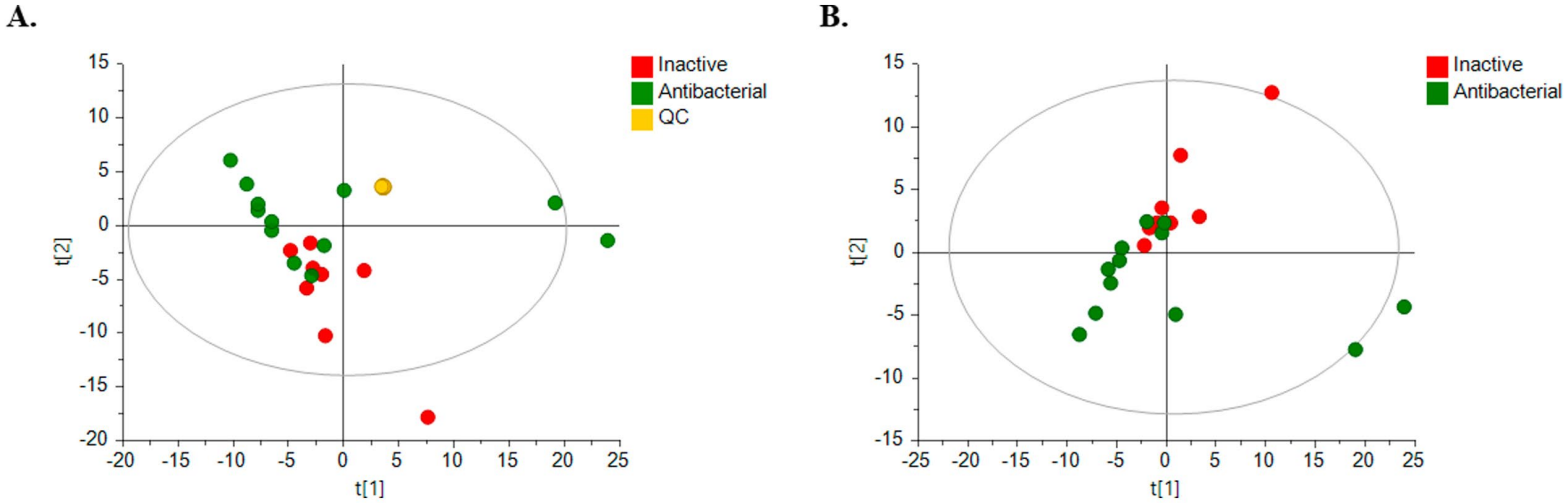
PCA score plots for untargeted metabolomics in positive ESI mode. (Green dots, bioactive samples; red dots, inactive samples; yellow dots, quality control samples, QC.) **(A)** HPLC-QTOF-MS (+) with QC samples: *R*^2^ = 0.733, *Q*^2^ = 0.293; **(B)** HPLC-QTOF-MS (+): *R*^2^ = 0.619, *Q*^2^ = 0.225.

Clear clustering of QC samples in the unsupervised PCA models (Figure 5A) showed the stability and quality of the acquired data for ESI analysis in the positive mode. Therefore, this result supports that separation between groups is related to biological differentiation. Additionally, PCA revealed distinct clustering of antibacterial (green) and inactive (red) samples (Figure 5B).

**Figure 6:**
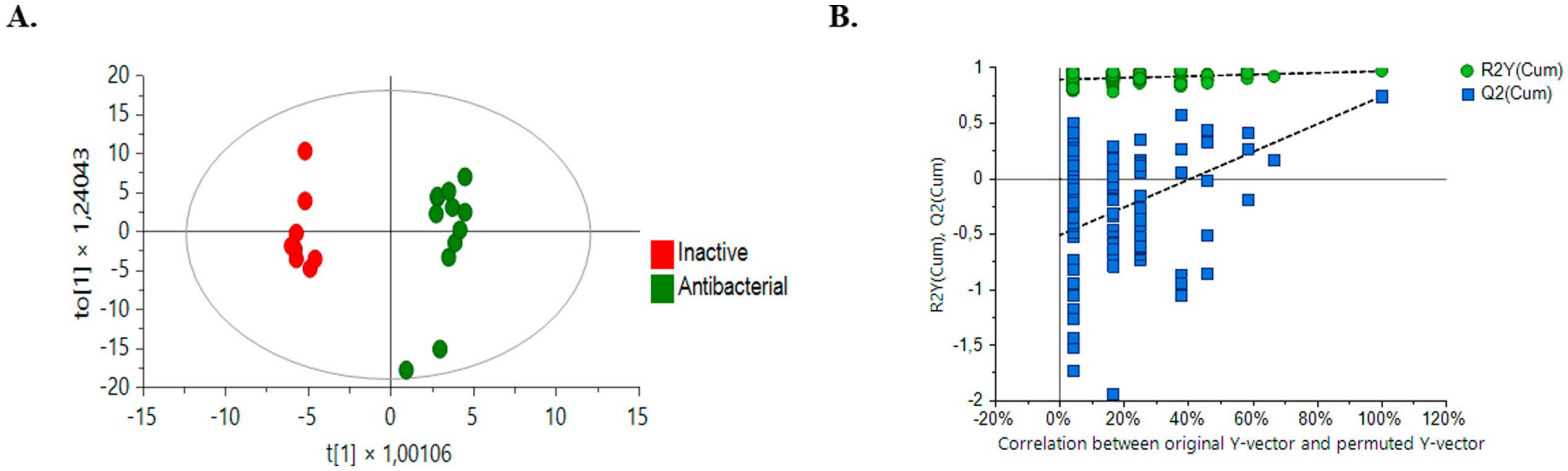
Comparison of supervised OPLS-DA models for untargeted metabolomics of inactive samples and bioactive samples (green dots, bioactive samples; red dots, inactive samples). **(A)** Positive ESI mode comparison HPLC-QTOF-MS (+): *R*^2^Y (*cum*): 0.966, *Q*^2^ (*cum*): 0.739, CV ANOVA: 1,3^–3^ **(B)** Permutation test (*n* = 100) results for the OPLS-DA model.

The OPLS-DA model successfully differentiated samples into active antibacterial and inactive extracts. The OPLS-DA score plots (Figure 6A) clearly show separation, with the active group represented by green circles and the inactive group represented by red circles. The quality and robustness of the OPLS-DA model were validated using permutation tests (*n* = 100) (Figure 6B). The *Q*^2^ intercept value was 0.739, indicating that the original model was statistically effective, the slope of the *Q*^2^ values in the permutation test was negative for Y axes, and all predicted *Q*^2^ values were lower than those calculated by the *Q*^2^ model, indicating not over-fitted model (Triba et al., 2015; Betancur et al., 2020).

A univariate analysis (UVA) was performed to assess the significance of each metabolite for comparison. The parameters used to select statistically significant metabolites were those that met the following criteria: *p* < 0.05, VIP >1. The metabolites that met these requirements were identified as putative, confirmed, or unknown and are presented in Table 2.

**Table 2:** Metabolites significantly differentiated between antibacterial active extracts and non-active extracts using ESI modes by untargeted metabolomics via quadrupole time-of-flight mass spectrometry.

										Antibacterial vs. inactive samples		
Compound	Formula	Mass	RT (min)	Mass Error (ppm)	Adduct	^a^CV for QC (%)^a^	Analytical platform	DET	ID level	^b^Fold change	^c^VIP	^d^*p*-value
Alkaloids												
Norharman	C_11_H_8_N_2_	168.0687	9.76	3	[M + H]^+^	1.13	GM-RF-LC-QTOF-MS	ESI+	2	3.77	1.43	9.56^−03^
Harman	C_12_H_10_N_2_	182.0844	10.76	3	[M + H]^+^	1.14	GM-RF-LC-QTOF-MS	ESI+	2	2.67	1.26	3.87^−02^
Hydroxyquinoline	C_9_H_7_NO	145.0528	6.88	3	[M + H]^+^	1.98	GM-RF-LC-QTOF-MS	ESI+	3	2.10	1.31	2.57^–02*^
Indoles and derivates												
Indole-carbinol	C_9_H_9_NO	147.0684	15.36	3	[M + H-H2O]^+^	6.60	GM-RF-LC-QTOF-MS	ESI+	2	2.54	1.38	7.30^−03^
Indoline	C_8_H_9_N	137.0841	14	5	[M + H-H2O]^+^	1.47	GM-RF-LC-QTOF-MS	ESI+	2	1.95	1.06	1.84^−02^
Fatty acyls												
Trimethyl-decatetraene	C_13_H_20_	176.1565	10.1	2	[M + Na]^+^	1.97	GM-RF-LC-QTOF-MS	ESI+	3	1.86	1.01	4.06^−02^
Benzene and substituted derivatives												
Di-tert-butylbenzene	C_14_H_22_	190.1722	12.44	4	[M + Na]^+^	1.90	GM-RF-LC-QTOF-MS	ESI+	3	1.96	1.08	4.06^−02^
Carboxylic acid												
Proclavaminic acid	C_8_H_14_N_2_O_4_	202.0954	2.3	2	[M + H-H2O]+	2.36	GM-RF-LC-QTOF-MS	ESI+	3	0.67	1.18	3.14^−02^
(Aminobutyl)-(hydroxy-methoxyphenyl)prop-enimidic acid	C_14_H_20_N_2_O_3_	264.1474	14	3	[M + H-H2O]+	1.39	GM-RF-LC-QTOF-MS	ESI+	3	2.05	1.12	2.26^−02^
Glycoside												
Chivosazole E	C_46_H_65_NO_12_	823.4507	18.83	5	[M + Na]^+^	9.75	GM-RF-LC-QTOF-MS	ESI+	3	0.04	1.75	9.56^−03^
Naphthalenes												
Desmethylterbinafine	C_20_H_23_N	277.183	15.36	3	[M + Na]+	4.63	GM-RF-LC-QTOF-MS	ESI+	3	2.26	1.13	2.02^−02^
Sphingoid bases												
Dimethyl-Safingol	C_20_H_43_NO_2_	329.3294	18.88	5	[M + H]^+^	4.67	GM-RF-LC-QTOF-MS	ESI+	3	0.26	1.52	4.58^–02*^
Sphingolipids												
SPB 18:0;O3	C_18_H_39_NO_3_	317.293	18.26	5	[M + H]^+^	2.34	GM-RF-LC-QTOF-MS	ESI+	3	0.19	1.69	2.57^–02*^
Stilbenes												
Longistylin A	C_20_H_22_O_2_	294.162	10.58	9	[M + H-H2O]^+^	1.28	GM-RF-LC-QTOF-MS	ESI+	4	2.31	1.30	2.02^−02^
Terpenoid												
Caryophyllene	C_14_H_22_	190.1721	10.94	5	[M + Na]^+^	1.57	GM-RF-LC-QTOF-MS	ESI+	3	3.85	1.55	2.01^−02^
Unknown	C_15_H_20_N_2_O_2_	260.1525	16.11	2	[M + H]+	2.09	GM-RF-LC-QTOF-MS	ESI+	4	2.14	1.44	2.52^−02^

^a^CV, coefficient of variation in the metabolites in the QC samples; ^b^Change, percent change in the abundance of the specified comparison calculated as ((case–control)/control) × 100, where the sign indicates the direction of change in the case group; ^c^VIP, variable importance in projection; ^d^*p*-value * corresponding to the *p*-values calculated by the Benjamini–Hochberg false discovery rate *post hoc* correction (FDR < 0.05). GM, global metabolomics; LC, liquid chromatography; QTOF-MS, quadrupole time-of-flight mass spectrometry. RF, reverse phase.

**Figure 7:**
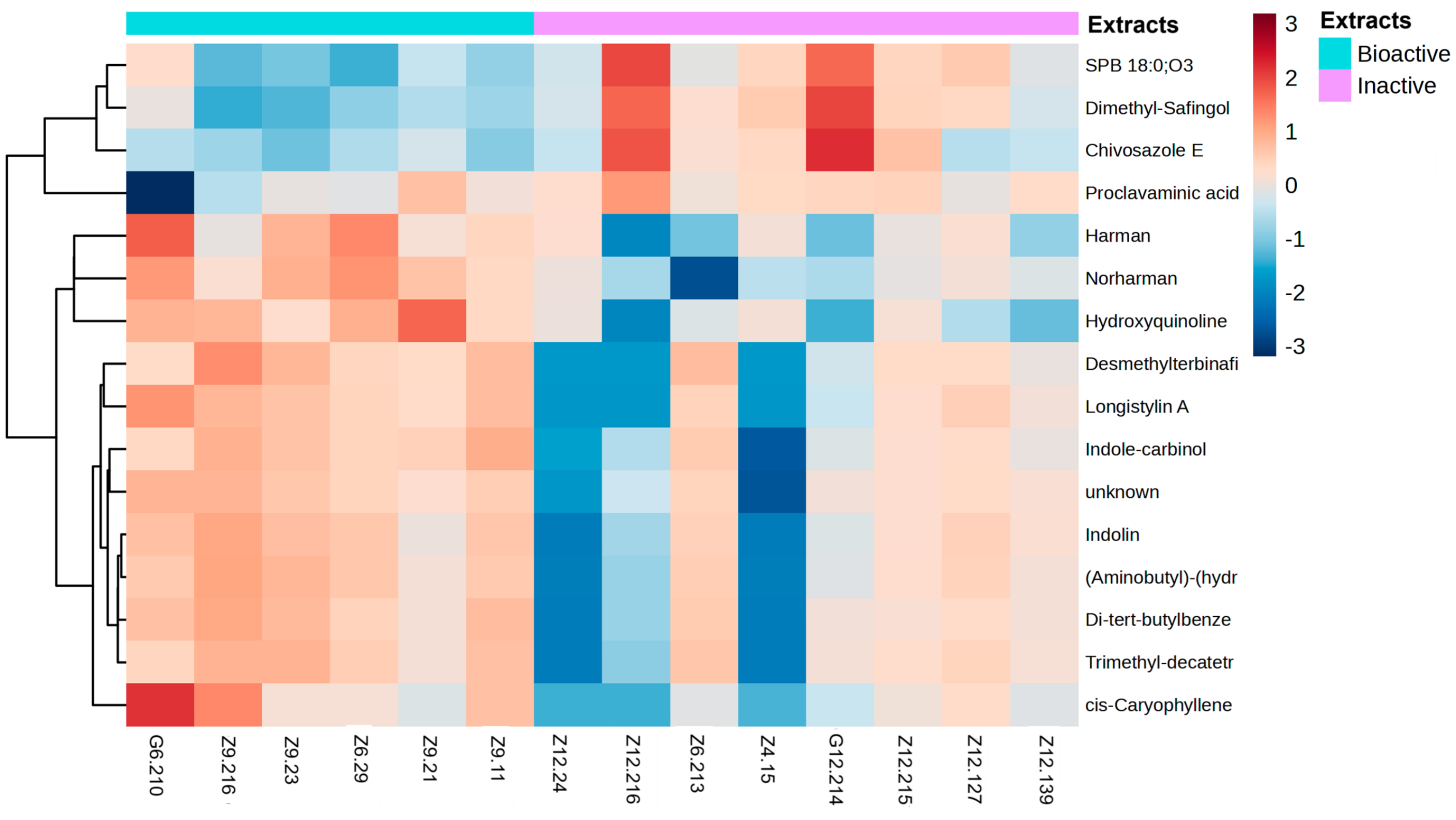
Heatmap of metabolites grouped by fold-change. The heatmap ranges from −3 to 3. Metabolites were identified by selecting the features most responsible for the antibacterial activity using discriminatory analysis (OPLS-DA), which were deemed significant by the linear model (*p*-value *<* 0.05). The antibacterial active extracts (denoted as G6.210, Z9.216, Z9.23, Z6.29, Z9.21, and Z9.11) and the non-active extracts (denoted as Z12.24, Z12.216, Z6.213, Z4.15, G12.214, Z12.215, Z12.127, and Z12.139). Hierarchical clustering of the strains was performed using Euclidean distances between the metabolites.

**Figure 8:**
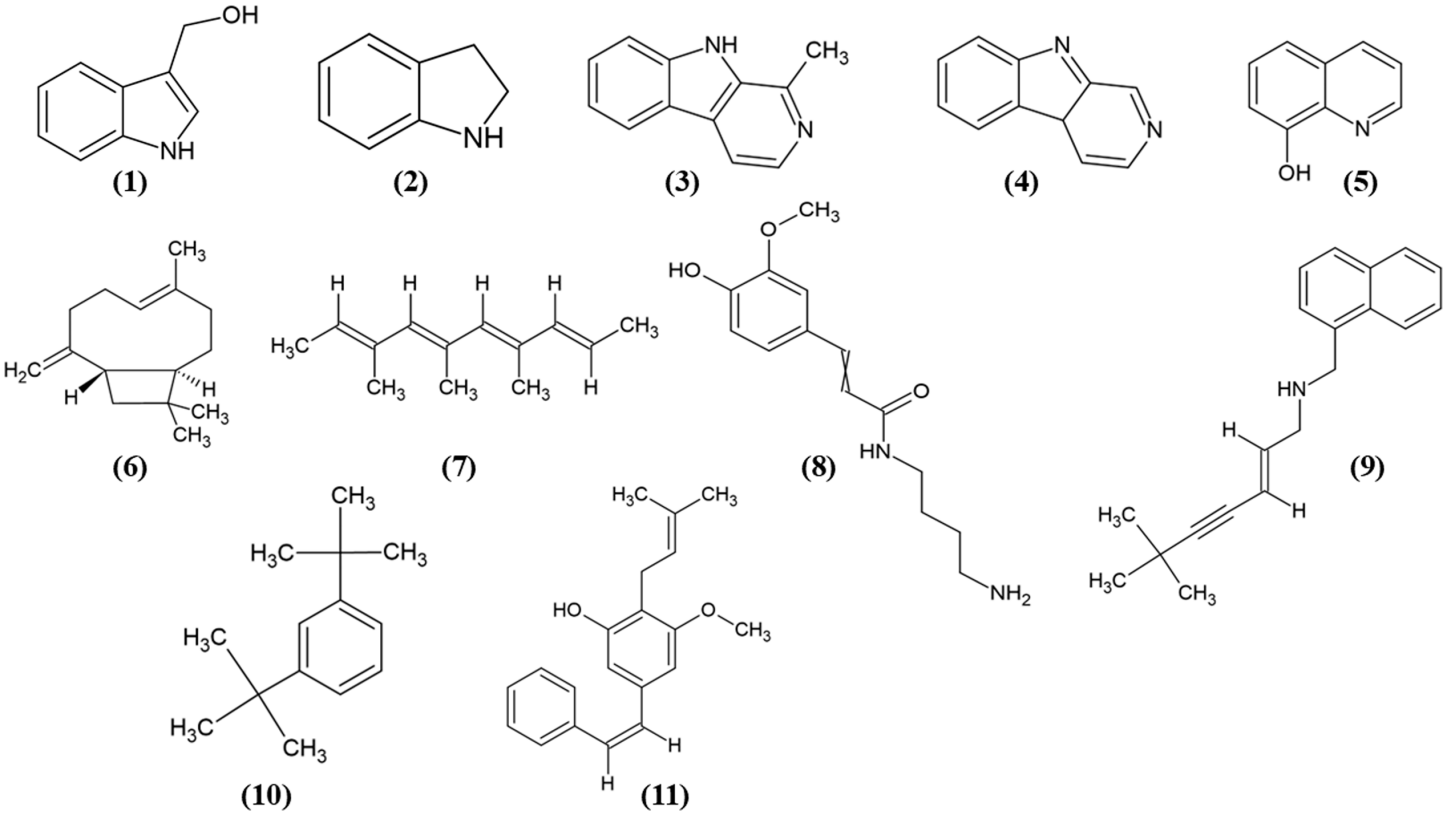
Chemical structures of the identified compounds at different confidence levels. Level 2 compounds (1–4): **(1)** Indole-3-carbinol, **(2)** Indoline, **(3)** Harman, and **(4)** Norharman. Level 3 compounds (5–11): **(5)** Hydroxyquinoline, **(6)** Caryophyllene, **(7)** Trimethyldecatetraene, **(8)** (Aminobutyl)-(hydroxy-methoxyphenyl) propenimidic acid, **(9)** Desmethylterbinafine, **(10)** Di-tert-butylbenzene, **(11)** Longistylin A.

Sixteen metabolites were identified as statistically significant when the antibacterial activities of the active and inactive extracts were compared. Among these compounds are alkaloids, such as indoles and their derivatives, quinolines, and other compounds, such as carboxylic acids, naphthalenes, stilbenes, and terpenoids (Figure 7). These features were characterized at different levels: level 2 (probable structure: matched to literature data or databases by diagnostic evidence) utilizing library spectrum matching through MS2 fragmentation with the aid of MSDial software and CFM-ID; level 3 (possible structure or class: most likely structure, isomers possible, substance class, or substructure match Probable structure: matched to literature data or database); and level 4 (an unknown feature of interest) based on the confidence level of compound annotation described by Blaženovi (2018). At level 2, we annotated four compounds (Figure 8), all of which belong to the alkaloid family. Notably, these compounds exhibited a high fold change (Table 2). One such compound, indole-carbinol **(1)**, has been reported in cruciferous vegetables and fungi (Lin et al., 2019; Sung and Lee, 2007). High-resolution time-of-flight mass spectrometry (HP*LC-QTOF-MS*) data for **(1)** showed adduct molecular ions at *m/z* 147.0684 [M + H-H2O]^+,^ which were analyzed using the molecular formula C_9_H_9_NO. Moreover, at Level 2, we annotated indoline **(2)**. HP*LC-QTOF-MS* data for **(2)** showed adduct molecular ions at *m/z* 137.0841 [M + H-H2O]^+,^ corresponding to the molecular formula C_8_H_9_N. This metabolite has been previously reported in actinobacteria with antibacterial properties, specifically in Streptomyces (Katsuyama, 2019). Harman **(3)**, a *β*-carboline alkaloid, was also annotated in this level of identification. HP*LC-QTOF-MS* data for **(3)** showed adduct molecular ions at *m/z* 182.0844 [M + H]^+,^ which were analyzed using the molecular formula C_12_H_10_N_2_. Finally, Norharman **(4),** another β-carboline alkaloid, was annotated with HP*LC-QTOF-MS* data, showing adduct molecular ions at *m/z* 168.0687 [M + H] ^+^, corresponding to the molecular formula C_11_H_8_N_2_.

Furthermore, we identified other compounds that have been mostly reported in Actinobacteria. Some of these compounds were hydroxyquinoline **(5)** and caryophyllene **(6)** (Figure 8). Similarly, we also annotated compounds at level 3 (Figure 8), although they have not been previously reported in actinobacteria, showed a great abundance (Table 2), some of which have been isolated from plants, such as trimethyl-decatetraene **(7)**, a fatty acyl compound, (aminobutyl)-(hydroxy-methoxyphenyl) prop-enimidic acid **(8)**, a compound belonging to the carboxylic acid class, which is a natural product of antimicrobial activity, desmethylterbinafine **(9)**, a naphthalene compound, di-tert-butylbenzene **(10)**, benzene and substituted derivative, and Longistylin A **(11),** a compound annotated at level 4, but this has been reported to have significant anti-MRSA activity (Wu et al., 2020) belonging to the Stilbenes class of compounds.

The remaining compounds, described in Table 2, were very low in abundance, especially in the inactive samples. Some of these compounds include clavaminic acid, carboxylic acid Chivosazole E, the glycoside compound dimethylsafingol, sphingoid base, SPB 18:0, and O3, a sphingolipid. Moreover, a considerable number of molecules were categorized as ‘unknown.’ However, among these, only one molecule met the criteria for statistical significance and demonstrated a biological relationship consistent with expected antibacterial activity.

## 4 Discussion

Actinobacteria are a relevant source of bioactive compounds primarily in terrestrial ecosystems (Kashfi et al., 2020). However, the repetitive discovery of land-based compounds (Ngamcharungchit et al., 2023; Siddharth and Rai, 2019) has allowed marine actinobacteria to emerge as promising alternative sources, leading to increased interest in marine bioprospecting. Corals, marine sponges, and their associated actinomycetes serve as repositories for novel natural products with diverse and potent biological activities and significant pharmaceutical value (Yang et al., 2015; Balasubramanian et al., 2018).

In our quest for new therapeutic options for acne vulgaris that minimize side effects and address bacterial resistance, we found that six out of 13 isolates studied could inhibit the growth of *S. epidermidis*, MRSA, and *C. acnes*. However, these extracts were characterized by a notably low yield, a challenge that has been previously described in similar studies (Lacret et al., 2019; Sujatha et al., 2005). Ethyl acetate was selected as the solvent because of its proven efficacy in isolating bioactive compounds from Actinobacteria, as documented in previous studies (Kurnianto et al., 2021; Sathish Kumar and Kokati Venkata Bhaskara, 2012). Remarkably, the Z9.216 extract displayed significant inhibition of over 95% against MRSA and *C. acnes,* and over 65% against *S. epidermidis* at a concentration of 0.003 mg/mL. The antibacterial activity of this extract was comparable to that of the standard antibiotic vancomycin, which was used as a reference, and exhibited 99% inhibition against both MRSA and *C. acnes* at the same concentration. These results align with those of previous studies, such as Paderog et al. (2020), who reported significant antibacterial activity of marine actinobacterial extracts against MRSA (Paderog et al., 2020).

Additionally, the growth rate assessment revealed no significant differences between the antibiotics and Z9.216 extract for MRSA and *C. acnes* (*p*-value < 0.05). Although research on the effects of actinobacterial extracts on *C. acnes* is limited, our findings are consistent with those of Kim et al. (2023) who demonstrated the antibacterial properties of a marine-derived pigment against *C. acnes*. The antibiotics currently used for *C. acnes* treatment, such as clindamycin and erythromycin, are derived from terrestrial actinobacteria, specifically from the *Streptomyces* genus, or are synthetically derived, such as tetracycline (De La Hoz-Romo et al., 2022). To the best of our knowledge, this study is among the first to demonstrate the therapeutic potential of marine actinobacterial extracts for treating acne vulgaris by targeting *C. acnes*.

Cytotoxic tests revealed that extract Z9.216 preserved the viability of human keratinocytes and fibroblasts at concentrations at which antibacterial activity was observed. In contrast, the Z9.23 extract caused the highest cytotoxicity at 1.25 mg/mL in HACAT cells, resulting in a viability reduction of up to 40%, a similar result also reported by Dahal et al. (2020).

*Streptomyces* remains the most prolific genus of actinobacteria (Newaz et al., 2022); however, bioprospecting of rare actinobacterial strains offers a promising source for identifying novel metabolites (Arasu Valan et al., 2016). In our study, four of the six bioactive strains belonged to the rare actinomycete strains *Kokuria* sp. strain EUFUS-Z9216*, Nocardia* sp. strain EUFUS-Z923, *Rhodococcus* sp. strain CLIVUS-Z629, and *Micrococcus* sp. strain EUFUS-Z911. Recent research has underscored the antibacterial potential of these less-known Actinobacteria genera. For instance, *Kocuria* species have been reported to produce antimicrobial compounds that are effective against various pathogenic bacteria, including MRSA (Jagannathan et al., 2021; Uzair et al., 2018). Similarly, *Nocardiopsis* strains have been found to synthesize novel antibiotics with potent activity against gram-positive and gram-negative bacteria (Kamarudheen et al., 2019). The diversity of rare actinobacterial strains examined in this study likely accounts for the range of compounds identified in the extracts.

Metabolomic analyses comparing antibacterial active and non-active extracts revealed diverse alkaloid derivatives as the dominant products, identified at level 2. While these compounds have predominantly been associated with the *Streptomyces* genus, as evidenced by the bulk of existing literature (Newaz et al., 2022; De Rop et al., 2022; Zhang et al., 2019; Yang et al., 2017; Zhang et al., 2017; Chen et al., 2019), their presence among extracts from other genera suggests a broader biosynthetic potential across various actinobacterial lineages.

In this study, a range of alkaloids were detected, including, hydroxyquinoline, derivatives of indole, Indole-carbinol, Indoline, and *β*-carboline compounds, such as Harman, and Norharman. These compounds were identified at the highest level and abundance, through metabolomic analysis as indicated by the fold change in Table 2. The indole nucleus, which is prevalent in many of these compounds, is a crucial structural motif in the pursuit of novel drug candidates and has been termed a “privileged structure” (Newaz et al., 2022; de Sa et al., 2009) owing to its versatile interactions with target proteins. Moreover, these compounds exhibit a spectrum of activities, including cytotoxic, antineoplastic, antibacterial, and antifungal (Newaz et al., 2022). In particular, the presence of nitrogen in their molecular architecture appears to play a crucial role in mediating these effects (Netz and Opatz, 2015).

Indole-carbinol, identified in this study, likely contributes to the antibacterial activity of bioactive extracts against MRSA, *S. epidermidis*, and *C. acnes*. This naturally occurring alkaloid, commonly found in cruciferous vegetables like broccoli and cauliflower (Netz and Opatz, 2015), is known for its anticancer, anti-inflammatory, and broad-spectrum antibacterial effects, including antibacterial activity against antibiotic-resistant strains (Sung and Lee, 2007; Wu et al., 2019; Wu et al., 2020).

Harman and Norharman, both β-carboline alkaloids, are commonly found in nature from a variety of sources, including plants, insects, and marine organisms (Netz and Opatz, 2015; Suzuki et al., 2018). Harman, known for its antibiotic potential, has previously been identified in marine invertebrates, notably within the tunicate-associated bacterium *Enterococcus faecium* (Aassila et al., 2003). This aligns with our findings as it supports the presence and potential bioactivity of similar compounds in marine actinobacterial extracts. Similarly, Norharman, an antimicrobial compound isolated from *Pseudoalteromonas piscicida* associated with the sponge *Hymeniacidon perleve*, was previously identified by Zheng et al. (2005) as a major antimicrobial agent against *Bacillus subtilis*, *S. aureus*, and *Escherichia coli* (Blockley et al., 2017). The β-carboline structure, common to both compounds, has been associated with the efficacy against certain antibiotic-resistant strains, suggesting its potential as a basis for novel antimicrobial agents (Suzuki et al., 2018). Additionally, the broad-spectrum antimicrobial activity of quaternary ammonium compounds against both gram-positive and gram-negative bacteria is noteworthy. These agents disrupt bacterial cell membranes by interacting with their negative charges, leading to the release of K+ ions and cytoplasmic content, ultimately causing bacterial cell death (Suzuki et al., 2018). Collectively, these insights help to elucidate our results and support the hypothesis that the observed antibacterial activity in actinobacterial extracts may be due to compounds with similar structures and mechanisms.

Among the identified metabolites, hydroxyquinoline (HQ), a quinoline-class alkaloid, demonstrated a statistically significant presence despite its low abundance in metabolomic analysis (Table 2). HQ is well known for its antimicrobial and anticancer activities, and has been frequently reported in actinobacteria, particularly within the *Streptomyces* genus (De Rop et al., 2022; Balthazar et al., 2022). The diverse biological properties of HQ and its derivatives, including anticancer, antibacterial, and anti-HIV activities, highlight their therapeutic relevance (Odingo et al., 2019; Song et al., 2015). HQ binds with a high affinity to various biological targets, offering the potential for new bioactive compound discovery (Song et al., 2015). The antibacterial mechanisms of HQ involve chelation with divalent ions, inhibition of RNA synthesis, and metallopeptidase activity. Structural modifications, particularly at positions 2 and 5, have been shown to enhance antibacterial efficacy, with substituted phenyl esters demonstrating potent activity against *S. aureus* and gram-negative bacteria. Notably, electron-withdrawing groups at the para position further increased this activity (Joaquim et al., 2021). The inclusion of HQ in our findings supports the hypothesis that such compounds contribute significantly to the antibacterial activity observed in marine actinobacterial extracts and underscores the potential for structural optimization to enhance bioactivity.

Additional identified compounds include caryophyllene, a sesquiterpene found widely in plants (Wu et al., 2020) and part of the terpenoid family, frequently reported in actinobacteria owing to terpene synthase genes in their genomes (Wu et al., 2020). Terpenoids exhibit various biological activities, such as antibacterial, antioxidant, anxiolytic, and anti-inflammatory effects, with caryophyllene also noted for its anti-aging and neuroprotective effects in animal studies (Rabe et al., 2013). Stilbenes and other compounds with high fold-changes, as shown in Table 2, are typical of actinobacteria and may contribute to the antibacterial activity of the extracts.

Four compounds were detected in the inactive samples, including two sphingolipids, which were significantly overproduced in these samples compared with the active ones. These sphingolipids displayed statistical significance, with adjusted *p-values* and high Variable Importance in Projection (VIP) scores, contributing to a clear separation between the groups. The excess production of these metabolites in the inactive extracts suggests a possible blocking effect of certain compounds related to antibacterial activity. Future studies could explore the impact of sphingolipids by adding them to active samples at varying concentrations to determine the point at which the antibacterial activity decreases. This approach could clarify whether the overproduction of these two metabolites was directly responsible for the observed reduction in activity in the inactive extracts.

This inhibitory role aligns with the known biological functions of sphingolipids, which are typically produced by eukaryotes but have been recently detected in some bacterial taxa (Kunz and Kozjak-Pavlovic, 2019). These lipids support membrane regeneration and may promote bacterial growth. Recent evidence has identified *Streptomyces aurantiacus* as being capable of synthesizing ceramides, which are fundamental components of more complex sphingolipids. Furthermore, sphingolipids can act as carbon sources, supporting bacterial growth and helping counteract antibacterial compounds that target cell membranes. This role could explain the probable antagonistic effects, emphasizing the need for further research on sphingolipids in bacterial metabolism and resistance mechanisms (Stankeviciute et al., 2019; Stankeviciute et al., 2022; Peters et al., 2024).

## 5 Conclusion and perspectives

Marine actinobacteria isolated from the sponge *Cliona varians* and the octocoral *Eunicea fusca* demonstrate considerable potential as a source of bioactive compounds effective against acne vulgaris-associated bacteria, including *Staphylococcus epidermidis*, methicillin-resistant *Staphylococcus aureus* (MRSA), and *Cutibacterium acnes*. The extract Z9.216 from *Kocuria* sp. exhibited significant antibacterial activity, comparable to that of conventional antibiotics, without cytotoxic effects on human keratinocytes and fibroblasts at effective concentrations. The identification of these bioactive strains, many of which belong to rare Actinobacteria, highlights an underexplored group with significant potential for novel therapeutic applications. Metabolomic profiling revealed diverse bioactive compounds, particularly alkaloids and terpenoids, which likely contribute to the observed antibacterial effects. Although identified at preliminary confidence levels, further structural elucidation using advanced techniques such as 1D and 2D carbon-hydrogen nuclear magnetic resonance (NMR) is necessary to confirm and detail these findings. Future research should also focus on optimizing the production methods for these potent extracts in marine actinobacteria and exploring delivery systems, such as encapsulation, to enhance stability and efficacy. Overall, our findings underscore the therapeutic potential of marine actinobacteria, positioning them as valuable sources for developing new and effective treatments for acne vulgaris, and laying the groundwork for further exploration and application of marine-derived bioactive compounds in dermatological health.

## Data Availability

The datasets presented in this study can be found in online repositories. The names of the repository/repositories and accession number(s) can be found in the article/supplementary material.

## Footnotes

^1^ https://www.combase.cc/index.php/en/

^2^ https://megasoftware.net
