# Supplementary material for: Marine actinobacteria metabolites: unlocking new treatments for acne vulgaris
Source: Front Microbiol. 2025 Jan 6;15:1501951. doi: 10.3389/fmicb.2024.1501951 (PMC11743623; doi:10.3389/fmicb.2024.1501951)
Supplement: Supplementary file 1 [file Table_1.DOCX]

**Supplementary material**

**Marine actinobacteria metabolites: unlocking new treatments for acne vulgaris**

María Clara De La Hoz-Romo^1,2^, Luis Díaz^1,2*^, Javier Gómez-León^3^, Marynes Quintero^3^, Luisa Villamil^1*^.

^1^Doctoral program of Biosciences, School of Engineering, Universidad de La Sabana, Chía, Cundinamarca, 140013, Colombia

^2^Bioprospecting Research Group, School of Engineering, Universidad de La Sabana, Chía 140013, Colombia

^3^Marine Bioprospecting Line, Marine and Coastal Research Institute “José Benito Vives de Andréis” INVEMAR, Calle 25 No. 2-55, Playa Salguero, Santa Marta D.T.C.H., Santa Marta, Colombia.

*** Correspondence:**Luisa Villamil
luisa.villamil@unisabana.edu.co

Luis Díaz

luis.diaz1@unisabana.edu.co

**Table S1:** Isolation sources of actinobacteria strains with antibacterial activity.

| No. | Strains code | Identification molecular strain | Isolation source | Type of animal | Database Link |
| --- | --- | --- | --- | --- | --- |
| 1 | Z9.216 | *Kocuria* sp. strain EUFUS-Z9216 | *Eunicea fusca* | Coral | <https://www.ncbi.nlm.nih.gov/nuccore/PP389604> |
| 2 | G6.210 | *Streptomyces* sp. CLIVUS-G6210 | Cliona varians | Sponge | <https://www.ncbi.nlm.nih.gov/nuccore/OK598074.1> |
| 3 | Z9.23 | *Nocardia* sp. strain EUFUS-Z923 | *Eunicea fusca* | Coral | <https://www.ncbi.nlm.nih.gov/nuccore/PP741801> |
| 4 | Z6.29 | *Rhodococcus* sp. strain CLIVUS-Z629 | Cliona varians | Sponge | <https://www.ncbi.nlm.nih.gov/nuccore/PP741804> |
| 5 | Z9.21 | *Streptomyces* sp. strain EUFUS-Z921 | *Eunicea fusca* | Coral | <https://www.ncbi.nlm.nih.gov/nuccore/PP741803.1> |
| 6 | Z9.11 | *Micrococcus* sp. strain EUFUS-Z911 | *Eunicea fusca* | Coral | <https://www.ncbi.nlm.nih.gov/nuccore/PP741802.1> |
